# Simulated leakage of high *p*CO_2_ water negatively impacts bivalve dominated infaunal communities from the Western Baltic Sea

**DOI:** 10.1038/srep31447

**Published:** 2016-08-19

**Authors:** Hanna Schade, Lisa Mevenkamp, Katja Guilini, Stefanie Meyer, Stanislav N. Gorb, Doris Abele, Ann Vanreusel, Frank Melzner

**Affiliations:** 1 GEOMAR, Helmholtz-Zentrum für Ozeanforschung Kiel, FB3/EOE-B, Hohenbergstr. 2, 24105 Kiel, Germany; 2 Marine Biology Research Group, Ghent University, Krijgslaan 281 - S8, 9000 Ghent, Belgium; 3 HGF-MPG Joint Research Group on Deep Sea Ecology and Technology, Alfred Wegener Institute for Polar and Marine Research, Am Handelshafen 12, 27570 Bremerhaven, Germany; 4 Max Planck Institute for Marine Microbiology, Celsiusstr. 1, 28359 Bremen, Germany; 5 CAU, Christian-Albrechts-Universitätzu Kiel, Zoological Institute: Functional Morphology and Biomechanics, Am BotanischenGarten 9, 24118 Kiel, Germany

## Abstract

Carbon capture and storage is promoted as a mitigation method counteracting the increase of atmospheric CO_2_ levels. However, at this stage, environmental consequences of potential CO_2_ leakage from sub-seabed storage sites are still largely unknown. In a 3-month-long mesocosm experiment, this study assessed the impact of elevated *p*CO_2_ levels (1,500 to 24,400 μatm) on *Cerastoderma edule* dominated benthic communities from the Baltic Sea. Mortality of *C. edule* was significantly increased in the highest treatment (24,400 μatm) and exceeded 50%. Furthermore, mortality of small size classes (0–1 cm) was significantly increased in treatment levels ≥6,600 μatm. First signs of external shell dissolution became visible at ≥1,500 μatm, holes were observed at >6,600 μatm. *C. edule* body condition decreased significantly at all treatment levels (1,500–24,400 μatm). Dominant meiofauna taxa remained unaffected in abundance. Densities of calcifying meiofauna taxa (i.e. Gastropoda and Ostracoda) decreased in high CO_2_ treatments (>6,600 μatm), while the non - calcifying Gastrotricha significantly increased in abundance at 24,400 μatm. In addition, microbial community composition was altered at the highest *p*CO_2_ level. We conclude that strong CO_2_ leakage can alter benthic infauna community composition at multiple trophic levels, likely due to high mortality of the dominant macrofauna species *C. edule*.

Carbon dioxide (CO_2_) is one of the most important natural greenhouse gases on Earth, playing a vital role in regulating the global heat budget and, thus, Earth’s climate system. Currently, the concentration of atmospheric CO_2_ is increasing at an unprecedented rate^1^, a process mainly caused by human actions^2^. Anthropogenic CO_2_, which enters the ocean, causes ocean acidification through an increase in dissolved inorganic carbon resulting in a decrease in pH. To reduce anthropogenic CO_2_ emissions to the atmosphere and mitigate climate change, the World Energy Outlook suggested long-term sequestration of CO_2_ (CCS) into sub-seabed geological structures as one suitable strategy^3^. Utilization of this technique, however, depends on the successful management of storage risks. These risks are still poorly constrained and current research is directed to obtain reliable information to establish a legal framework for environmentally safe application of CCS and efficient monitoring techniques^4^,^5^.

One of the main potential risks of CCS is CO_2_ leakage through e.g. fracture zones or pipe failure leading to acidification of the pore and bottom water or water column, respectively^6^. The extent of impacts of acidified seawater on the surrounding marine ecosystem will depend on the scale and intensity of leaks, the local hydrodynamic regime and the duration of exposure^7^. Models that evaluated different potential leakage scenarios in the southern part of the North Sea predict temporary local seawater pH changes ranging from <−0.12 in case of a continuous diffuse seepage up to >−1.0 pH units in a continuous point source leakage scenario^7^,^8^. The latter represents the extreme case of a major geological fault break or an unrecognized pipe failure with outgassing of high amounts of CO_2_ into the marine environment^9^,^10^,^11^,^12^. Experimental release of ca. 4 tons of CO_2_ via sediment injection during the 37 day long QUICS experiment in a Scottish Sea Loch resulted in maximum decreases in sediment pH by >0.8 pH units^12^.

Studies that have investigated the influence of elevated seawater *p*CO_2_ on benthic organisms primarily used short- to medium-term exposures, single species experiments and *p*CO_2_ levels relevant for ocean acidification (OA) forecasts. The influence of medium-term exposure to high *p*CO_2_ on communities is not well investigated and different threshold levels need to be identified. The sensitivity of marine organisms to varying *p*CO_2_ levels and accompanying seawater pH variations differs between taxa, resulting in very diverse responses to elevated internal CO_2_ concentrations (hypercapnia)^13^. The general response of animals to hypercapnia is related to disturbance and regulation of the intra- and extracellular acid-base balance^13^,^14^,^15^. Those processes are energy consuming and can result in growth reduction, decreased metabolic activity, low reproduction rates, behavioural changes or ultimately death^13^,^16^,^17^. Calcifying organisms are particularly susceptible to acidified seawater due to reduced aragonite and calcite saturation of the water which can lead to enhanced dissolution of unprotected skeleton components and reduced availability of inorganic carbon for calcification^18^,^19^,^20^. Non-calcifying, infaunal invertebrates (e.g. meiofauna, and more particular the dominant nematodes) with a low mobility and bacterial communities are naturally exposed to large fluctuations of pore water pH and *p*CO_2_^21^,^22^, and are therefore likely to be more tolerant to high *p*CO_2_ (OA)^13^.

Only a handful of studies have exposed sediment communities in their natural composition to elevated seawater *p*CO_2_, primarily due to the great logistic effort necessary to collect and maintain such communities in the laboratory and to control the carbonate system with sufficient accuracy during experiments. Negative effects of medium-term (20 weeks) exposure to high seawater *p*CO_2_ (pH 7.3–5.6) have been reported on macro- and meiofauna diversity and community structure^23^. In addition, Widdicombe and Needham^24^ could show that even though seawater acidification did not alter nereid worm burrow size or structure, sediment nutrient fluxes were changed after five weeks of acidification exposure, supposedly due to changes in bacterial communities. Moreover, since the burrowing activity of e.g. echinoderms has a strong influence on the biogeochemistry of sediments and the composition of meiofauna communities^25^, it seems likely that changes in macrofauna abundance in response to elevated seawater *p*CO_2_ can have strong repercussions on infaunal ecosystem processes^23^,^24^.

Up to date, no comprehensive study has addressed the impact of elevated seawater *p*CO_2_ and acidification on benthic bivalve dominated communities (e.g. bivalves, meiofauna and bacteria) thriving in shallow, sandy coastal sediments in the North Atlantic region. Bivalves are of high ecological importance as they represent the defining macrobenthic organisms in these habitats, with a key nutritional role for many fish and migratory bird species^26^,^27^. As they filter large amounts of water, deposit particulate organic matter onto and into the sediment and provide settlement habitat for other organisms, they are irreplaceable key players in the benthic-pelagic coupling^28^,^29^.

A range of recent studies on several (mainly epibenthic) bivalve species could establish that calcification, growth, filtration and metabolism can be negatively impacted by elevated seawater *p*CO_2_, often already at moderately decreased pH >7.5 (ca. <5,000 μatm). In addition, increased occurrence of oxidative and acid-base regulatory stress has been observed in bivalves^16^,^30^,^31^,^32^. Detrimental external and internal shell dissolution occurs in several species when these are exposed to acidified seawater for prolonged time intervals (weeks), with less severe effects observed in species that possess a thick and intact periostracum^33^,^34^,^35^,^36^. However, biological impacts such as bioturbation^22^,^24^,^25^ or interactions between and among different taxa and trophic groups^23^,^25^,^37^,^38^ can result in very diverse responses of the biological community when compared to single species/taxon studies. Mesocosm experiments which address the effect of seawater acidification on shallow water benthic species report an overall higher sensitivity of macrofauna while meiofauna, with nematodes as dominant and commonly reported taxon, remain unaffected or even increase in abundance^23^,^25^,^37^,^39^. Laboratory experiments confirm a strong resistance of dominant meiofauna taxa to pH changes as expected to occur as a result of OA by the year 2300^39^ but a reduced survival of nematodes has been observed when the pH is further reduced to values that could potentially occur in response to a severe CCS leakage scenario (<pH 7)^40^. Microbial communities are important for the coastal ecosystem as they are primarily responsible for organic matter remineralisation, nutrient cycling and function as food source for grazing marine organisms^41^,^42^,^43^,^44^. Investigations about the direct effects of lowered pH are still inconsistent^41^,^45^. While some studies observed differences in abundance of a few genera or rare taxa, others found a change in bacterial biomass production with increasing *p*CO_2_^46^,^47^,^48^,^49^.

In this study, we conducted a mesocosm experiment using natural *C. edule* dominated sandy communities from the Western Baltic Sea utilizing a flow-through seawater design with optimized food supply. Three of the dominant infauna bivalve species from the western Baltic and North Sea are the cockle *Cerastoderma edule*, the soft-shell clam *Mya arenaria,* and the Baltic tellin *Macoma balthica*^50^. *C. edule* and *M. balthica* live within the top two to five centimetres of the sediment, while *M. arenaria* occurs to sediment depths of up to 50 cm^51^. Generally, all three species are widespread in tidal flats and shallow coastal areas and serve as an important link between primary producers and consumers. Owing to their very high densities in the sediment (often >1,000 individuals m^−2^) and considerable mobility and activity^52^ they exert a strong influence on infaunal communities and biogeochemical processes, e.g. by increasing primary production and fertilization of microphytobenthos through NH_4_^+^ excretion^52^,^53^. To predict marine benthic ecosystem vulnerability to potential chronic leakages from CCS storage sites, experiments need to utilize a high *p*CO_2_ range, realize a multispecies approach and intermediate exposure durations. In search for an indicator species useable in future CCS site assessments, experiments focused on the most abundant bivalve *C. edule*. We hypothesized that *C. edule* would be very sensitive to elevated *p*CO_2_, as it lives close to the sediment surface. *C. edule* mainly occurs in relatively unpolluted areas while e.g. *M. balthica* can also be found in polluted and hypoxic areas^54^, suggesting a higher resistance of *M. balthica* to unfavourable environmental conditions. To extend the common single-species approach to an ecosystem investigation and to test for the role of *C. edule* as an important ecosystem engineer, the impact of high *p*CO_2_ on benthic meiofauna and bacterial communities was also assessed. We hypothesized that elevated *p*CO_2_ and changes in bivalve abundance and fitness would impact meiofaunal and microbial communities.

## Materials and Methods

### Experimental setup

Sandy communities were exposed to six different seawater *p*CO_2_ regimes for a total of three months (17.12.2011–06.03.2012) in a climate - controlled room. Six header tanks were continuously supplied with filtered seawater from Kiel Fjord, each one connected to six experimental units (EU) ensuring continuous seawater supply (Supplementary Figure S5). This design is a randomized design (B4) according to Cornwell and Hurd^55^. Each EU consisted of a round plastic container with a volume of 12.5 L containing ca. 9.5 L of sediment and an overlying water column of ca. 3 L. The lower 10 cm of the sediment consisted of sieved sand taken from a local beach (Kiel, Falckenstein: 54°23,66 N; 10°11.56 E) while the upper 10 cm consisted of surface sediment from the station at which the experimental animals were sampled to resemble natural conditions as well as to provide naturally occurring microbial and meiofauna communities. Bivalves and sediment were sampled in Kiel Fjord at Falckenstein with a Van Veen grab in 1–2 m depth using the vessel FK Polarfuchs on November 21^st^ 2011 and kept in holding basins at 9 °C before being placed in EUs. Density of infauna bivalves was determined during the sampling process. 1 m^2^ of sediment at Falckenstein was found to contain 146 *M. arenaria*, 9 *M. balthica,* and 1,040 *C. edule*. In order to simulate a natural size distribution in our laboratory experiment, we sieved the collected sediment (1 mm mesh width). All bivalves were taken out and replaced by a defined number per EU: 5 *M. arenaria* (size classes: 0.5–1 cm: 2 animals; 1–1.5 cm: 2 animals; 2–2.5 cm: 1 animal), 1 *M. balthica,* and 40 *C. edule* (size classes: 0–0.5 cm: 3 animals; 0.5–1 cm: 18 animals; 1–1.5 cm: 11 animals; 1.5–2 cm: 7 animals; 2–2.5 cm: 1 animal). Small gastropods (exclusively *Hydrobia* spp.) were abundant with ~10 individuals per EU. Due to their small size (<0.5 mm) they were randomly distributed within all EUs with the sieved sediment. Due to the natural low diversity of the Baltic, the density of other macrofauna individuals was <1 individuals per m^2^. These low abundant species (e.g. nereid polychaetes, pharid bivalve species) were excluded from the experiment. The experimental units were kept in a seawater flow-through system for two weeks under control conditions prior to the experiment to allow proper acclimatization of biogeochemistry and the faunal community. Seawater pH was maintained in the header tanks using a pH feedback system (IKS Aquastar, iksComputersysteme GmbH, Karlsbad, Germany). Treatment levels were achieved through continuous addition of acidified water from the header tanks into the overlaying seawater of each EU and included levels of 900 μatm (control, pH 7.8 NBS scale), 1,500 μatm (pH 7.7), 2,900 μatm (pH 7.4), 6,600 μatm (pH 7.0), 12,800 μatm (pH 6.7), and 24,400 μatm (pH 6.4) (Supplementary Table S1). 900 μatm was used as a control due to the high background *p*CO_2_ in Kiel Fjord^31^,^35^. To support the bivalve nutritional needs unicellular algae (*Rhodomonas* sp.) were cultured as described in Thomsen *et al*.^35^ and added continuously into the header tanks via a peristaltic pump, thus maintaining a stable concentration of 3,500–4,000 (Supplementary Table S1) cells ml^−1^ within header tanks (Supplementary Table S1).

A flow rate of 100 ml min^−1^ was provided to each EU from the respective header tank via gravity feed. Throughout the experiment, pH, salinity, temperature, and flow rate were measured daily in each replicate. Salinity and temperature fluctuated in accordance with naturally occurring changes in Kiel Fjord seawater (14.6–20.5 psu and 4.3–8.9 °C, respectively). Light conditions were similar for all EUs. Dead animals were removed daily and behaviour of bivalves (presence/absence on the sediment surface) was noted every other day starting in the third experimental week. Carbonate chemistry and algae concentration in the EUs were measured weekly. Dissolved inorganic carbon (*C*_T_) was measured using an Automated Infrared Inorganic Carbon Analyzer (AIRICA, Marianda, Kiel, Germany). Seawater chemistry (*p*CO_2_ and calcium carbonate saturation state) was then calculated according to the guide to best practices for ocean CO_2_ measurements^56^, using CO2SYS^57^ with pH (NBS scale) and *C*_T_, temperature, salinity, and first and second dissociation constants of carbonic acid in seawater^47^.

### Bivalve sampling and processing

At the end of the experiment, all but four *C. edule* specimen per EU were frozen at −20 °C. Shell-free dry mass was measured according to Thomsen *et al*.^31^. To survey shell dissolution, five randomly selected *C. edule* from each treatment were analyzed using a stereomicroscope (40-fold magnification). In addition, scanning electron microscopy (SEM) was used to examine external shell dissolution at a higher resolution. For that purpose, three shells of the 900–6,600 μatm CO_2_ treatments (as signs of dissolution were obvious at higher levels) were mounted separately on SEM pedestal stubs, coated with gold-palladium and examined using scanning electron microscopy (Nanolab 7, Zeiss, Oberkochen, Germany and HitachiS4800, Hitachi High - Technologies Europe, Krefeld, Germany).

The extent of oxidative stress in the lipid fraction of the whole body tissue was estimated by analysing the malondialdehyde (MDA, a marker of lipid peroxidation) content in four *C. edule* specimens per EU, which were stored frozen at −80 °C prior to measurement. The entire tissue mass of each bivalve was separately processed via grinding in liquid nitrogen. Fifty milligrams of ground tissue powder of each of the four specimens from the same EU were pooled together to obtain sufficient tissue amounts for the analysis (one pool per EU). MDA concentration of each replicate was determined following the protocol of Uchiyama and Mihara^58^. Approximately 100 mg tissue-powder was homogenized with 0.2% phosphoric acid in a 1:5 ratio (sample/phosphoric acid). In the next step an equivalent volume of 2% phosphoric acid was added. One blank (homogenate + 0.2 ml 3 mM hydrogen chloride) and two samples (homogenate + 0.2 ml TBA solution) were adjusted to a pH of 1.6 (with HCl or NaOH) and were incubated at 100 °C for one hour. After cooling, 0.5 ml of butanol was added, samples were vortexed for 40 seconds and centrifuged at 1,000 g for 5 minutes. The supernatant was collected into fresh vials and centrifuged for 5 minutes at 14,000 g. Samples and blanks were transferred to a 96 well plate and the extinction was measured in a plate reader (Plate Chameleon, Hidex, Turku, Finland) at 532 and 600 nm. Tissue concentration of MDA was calculated following equation (1).


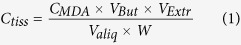


C_MDA_: MDA concentration

V_But_: volume of butanol [ml]

V_Extr_: extraction volume [ml]

V_aliq_: volume of homogenate [ml]

W: weight of tissue [g]

### Meiofauna sampling and processing

The uppermost centimetre of each EU was sampled after six and twelve weeks using a small corer (Ø 2.5 cm) and stored in 4% buffered paraformaldehyde until further extraction of the meiofauna. For this purpose, samples were washed on two stacked sieves in order to separate the macrofauna fraction (on a 1mm sieve) from the meiofauna fraction (on a 38 μm sieve). Subsequently, meiofauna was extracted by triple density gradient centrifugation (3000 rpm) with colloidal silica gel LUDOX HS40 Dupont (specific gravity 1.18) as a flotation medium^59^, fixed in 4% buffered formaldehyde solution and stained with Rose Bengal. Meiofauna was counted under a stereo microscope (50x magnification) and identified to higher taxonomic level consulting e.g. Higgins and Thiel^60^. Only nematodes that were in a good shape and showed no clear signs of degradation (loose cuticle, lack or damage of internal structures, biofilm, flattened or dehydrated habitus) were counted. For nematode identification, 100 nematodes were picked out per sample, transferred to glycerine following the protocol of De Grisse^61^ and subsequently mounted on paraffin-ring glass slides. Nematodes were identified to genus level following a pictorial key^62^ and the NeMys database^63^ under a microscope (Leica DMR, 10x–100x magnification).

### Microbial community sampling and processing

After six and twelve weeks, one corer sample (Ø 2.5 cm, 1 cm depth) per EU was collected, transferred into 50 ml plastic tubes and stored frozen at −20 °C for bacterial community analysis. The samples were subjected to total community DNA extraction using the FastDNA SPIN Kit for Soil (Qbiogene, Carlsbad, CA) including an additional heating step to increase yield and final elution of the DNA in TE-buffer. Benthic bacterial community structures were determined by means of the high-throughput fingerprinting technique ARISA, following a previously published procedure by Ramette^43^ with slight modifications: Final concentrations of PCR ingredients within 50 μl-reactions were 0.4 μM of each primer, 0.1 mg ml^−1^ BSA, 250 μM of each dNTP (peqGOLD Kit; Peqlab, Erlangen, Germany), 1x Buffer S with 1.5 mM MgCl_2_(Peqlab), 1.0 mM extra MgCl_2_ (Peqlab) and 2.5 U peqGOLD*Taq*-DNA-Polymerase (Peqlab). The forward primer was labelled with FAM at its 5′-end. For each sample (EU), three PCR replicates were prepared. For two samples (EUs) a successful amplification could not be validated, they were therefore excluded from further analyses.

### Statistical analyses

Univariate, parametric and non-parametric analyses were performed using the program R version 3.0.2.^64^. Multivariate, non-parametric analyses were performed with the program Primer version 6.1.11 with the add-on software Permanova+ version 1.0.1^65^,^66^. Regression analysis (ANCOVA) was performed using Prism 6 software (GraphPad Software, Inc.). A significance level of α = 5% was chosen for all statistical tests. Significant test results are reported in the results, statistical information can be found in the supplementary tables.

To test for differences in *C. edule* behaviour, a permutational ANOVA (PERMANOVA), with the factor EU nested in treatment, was used as a non-parametric solution to a repeated measures analysis. *C. edule* mortality between treatments and the fraction of dissolved shells between treatments was tested using a Kruskal-Wallis test combined with a Kruskal multiple comparisons test since assumptions of normality (Shapiro-Wilk test) and homogeneity of variances (Levene’s test) were not met. Mortality between different size classes within each treatment was tested with a Kruskal-Wallis test. For this, animals were pooled in two groups, large (1–2, 5 cm shell width) and small (0–1 cm shell width). The influence of *p*CO_2_ on malondialdehyde (MDA) content was tested using a one-way analysis of variance (ANOVA) and a Tukey HSD post hoc test. The difference in shell-free dry mass between treatments was tested with an analysis of covariance (ANCOVA), using log shell - free dry mass and log shell width. As no significant interaction was found for replicate and treatment (nested ANOVA, EU nested in CO_2_ treatment, p-value = 0.8411), all measured animals (n = 479) of each treatment were used in the regression analysis.

Meiofauna community analysis was based on calculated densities (individuals 10 cm^−^^2^) while nematode community analysis was based on relative abundances and calculated densities. Due to an unbalanced and non-normal distributed dataset, Permanova was chosen as an ANOVA approach for repeated measures to test for differences in total meiofauna densities and meiofauna and nematode community structure between treatments, time and interaction of both, with EU nested in treatment. When the number of unique permutations was lower than ten a Monte Carlo (MC) test was applied to calculate the p-value. The data were square root transformed and Bray-Curtis resemblance matrices were calculated. A pairwise-comparison test was executed for factors that differed significantly. The PermDisp tests always assured homogeneity of data dispersion unless mentioned otherwise. A multidimensional scaling (MDS) plot was created to visualize the results. Permanova was repeated for the meiofauna dataset after excluding Nematoda and a SIMPER analysis was performed to reveal the taxa that contributed most to the differences between treatments. The taxa with the most impact on dissimilarities were tested each by a univariate Permanova analysis as described above. For taxa with empty samples a dummy variable was added to avoid undefined values when calculating the Bray-Curtis resemblance matrix as suggested by Clarke *et al*.^67^. Meiofauna diversity indices (Shannon-Wiener index, Simpson index and Pielou’s evenness index) were calculated and tested for significant differences between treatments with Permanova.

Quality assessment of raw bacterial community profiles and binning (2–3 replicates per sample) were done as previously reported^43^. Merged community profiles were generated in R (v.2.13.2^64^); by using a custom script and considering Operational Taxonomic Units (OTUs) that occurred at least twice^43^. Non-metric Multidimensional Scaling (NMDS) was used to represent dissimilarity matrices based on Bray-Curtis or Jaccard coefficients into a reduced space^68^. Analysis of similarity (ANOSIM) was conducted with the PAST software (Version 1.76^69^). To test for the effect of *p*CO_2_ treatment and time on bacterial community composition, multivariate ANOVA and variation partitioning were conducted in R.

## Results

### Bivalve community response

**Figure 1:**
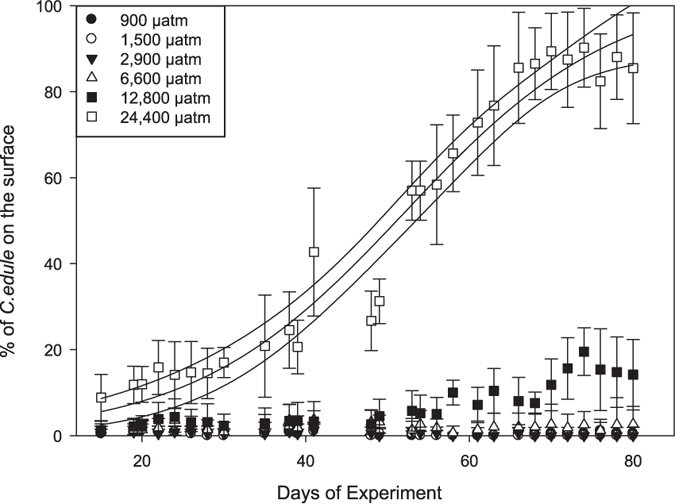
*C. edule* behaviour. Average abundance of non-buried *C. edule* over the complete experimental phase in % of total, curve fitted for 24,400 μatm including 95% confidence interval.

Out of the three investigated bivalve species, the cockle *C. edule* was the most abundant and most sensitive to acidified seawater. As the abundance of the other two bivalve species was very low and no mortality was recorded, we focus on *C. edule* in the following sections. Throughout the experiment, cockles were either buried with open siphos or lying on the sediment surface. Under control conditions and at 1,500 μatm, cockles were mostly buried (Fig. 1). In the highest CO_2_ treatment (24,400 μatm), cockles migrated towards the surface (Fig. 1). At 12,800 μatm, some cockles were observed on the surface, however always at a lower abundance than in the highest treatment. The distribution of cockles on the surface was significantly influenced by CO_2_ treatment (p(perm) = 0.0001; Supplementary Table S2). Although time and the interaction between time and treatment were significant (p(perm) = 0.0001), 63% of the variance were explained by the factor treatment. In the 24,400 μatm treatment, 50% of cockles were located on the sediment surface on day 50 (Fig. 1).

**Figure 2:**
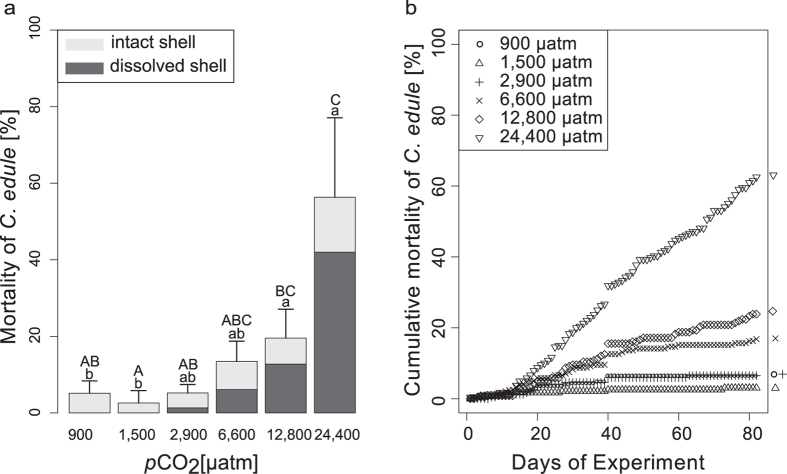
(**a**) Bars showing *C. edule* mortality over the entire experimental duration (mean ± SD); colour coding showing fraction of corroded vs. intact shell (white = intact, grey = corroded); letters indicate significant differences between treatments. (**b**) Cumulative mortality plotted over the duration of the experiment. 50% mortality at 24,400 μatm was reached at day 68.

**Figure 3:**
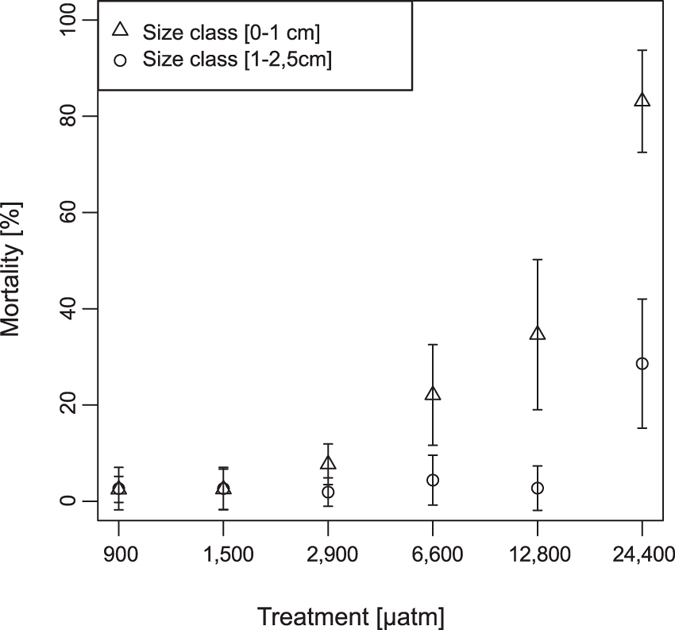
Cumulative mortality of different *C. edule* size classes during the experiment plotted for each treatment.

Mortality of *C. edule* increased with seawater *p*CO_2_ with 50% mortality in the highest treatment already after 68 days (Fig. 2). A significantly elevated mortality could be shown for the 24,400 μatm group compared to the control, 1,500 μatm, and 2,900 μatm groups (Kruskal-Wallis multiple comparison test, p < 0.05, Supplementary Table S3, S3a). Mortality, when averaged over all size classes, tended to increase in 12,800 μatm as well (total mortality ca. 15%). However, this increase was not significant by the end of the experiment. Smaller individuals reacted more sensitively towards high *p*CO_2_ (Fig. 3). There were no differences in mortality between size classes in the control, 1,500 μatm, and 2,900 μatm treatments. At 6,600, 12,800 and 24,400 μatm, mortality in the smaller size class (0–1 cm) was significantly higher than mortality of cockles in the larger size class (1–2.5 cm) (Kruskal-Wallis multiple comparison test, p < 0.05, Supplementary Table S4).

**Figure 4:**
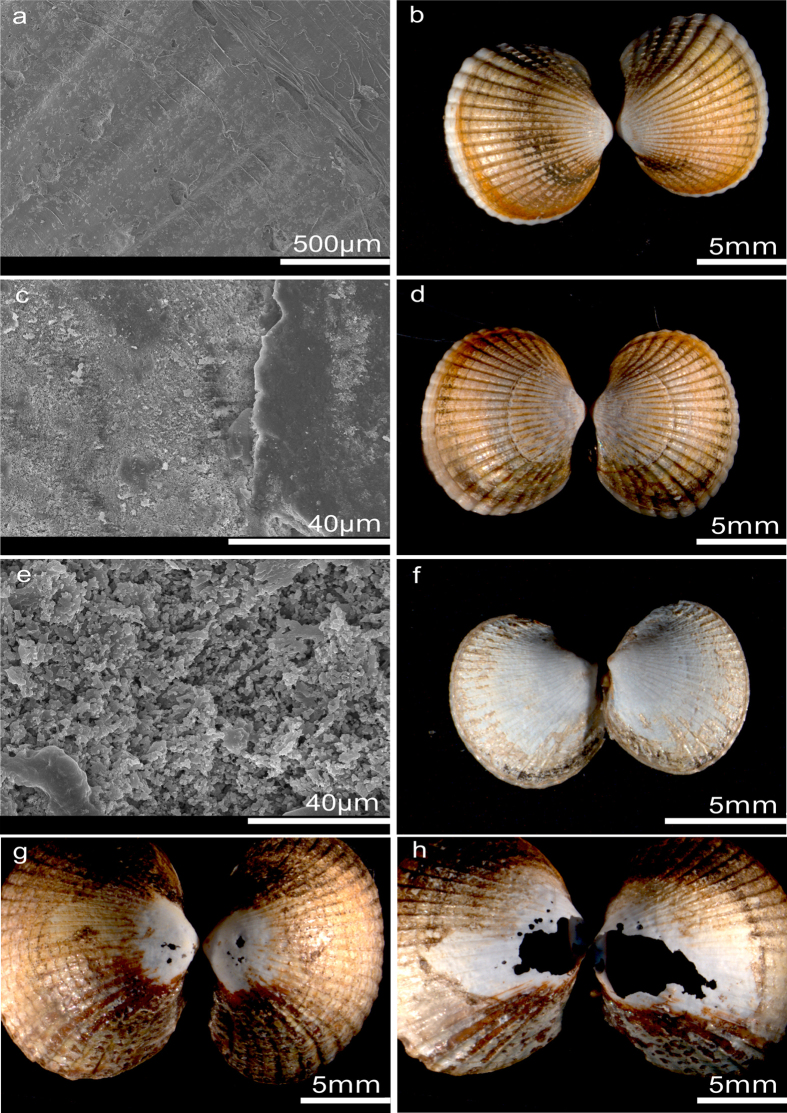
Shell corrosion in *C. edule*. (**a,b**) Control (900 μatm), no shell corrosion visible on the outside of the shell. (**c,d**) 1,500 μatm, shell corrosion on the outside of the shell. (**e,f**) 6,600 μatm, shell dissolution on the outside of the shell. (**g,h**) 24,400 μatm, strong dissolution signs, holes.

The comparison of intact shells against shells with signs of dissolution showed an increase in shell corrosion of *C. edule* with increasing *p*CO_2_ (Figs 2 and 4). Shells from the control treatment had an intact periostracum and were not characterized by shell dissolution (Fig. 4, N = 3 of 3 observations). Shells from ≥1,500 μatm were characterized by signs of external dissolution (SEM analysis, N = 3 of 3 observations). Stereo microscopic images demonstrated visible signs of shell dissolution for all treatments above 2,900 μatm, with increasing severity in higher *p*CO_2_ treatments (Fig. 4). For some cockles (<5%), exclusively small animals (<0.7 cm) holes were already visible at 2,900 μatm. Cockles maintained under high *p*CO_2_ (6,600, 12,800 and 24,400 μatm) were characterized by severe shell damage. In the 6,600 μatm treatment, 72% of dead cockles, mostly small individuals, contained holes in their shells. In the two highest treatments (12,800 μatm and 24,400 μatm) >85% of deceased cockles were characterized by holes in their shells. This rate of shell dissolution was significantly higher when compared to the control and 1,500 μatm treatment (Kruskal-Wallis, p < 0.05, Supplementary Table S3, S3b).

**Figure 5:**
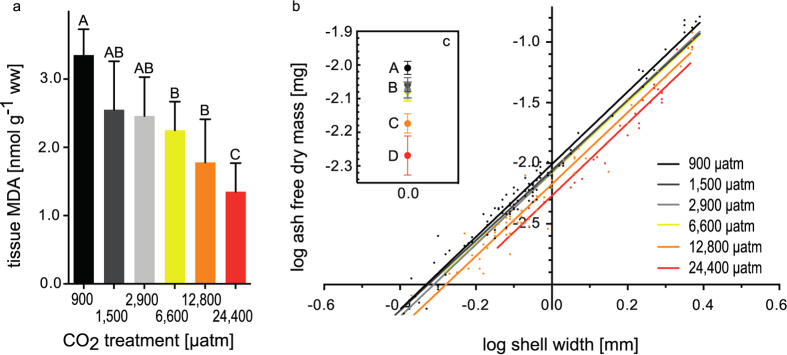
*C. edule* condition and MDA accumulation. (**a**) Average of tissue MDA content [nmol g^−1^ww] for the different treatments. Means and standard deviation, letters indicate significant differences between treatments. (**b**) Regressions of log shell-free dry mass plotted against log shell width, single data points plotted for the 900, 12,800 and 24,400 μatm treatment (equations in Supplementary Table S5) (**c**) y intercepts of regressions of shell free dry mass and 95% confidence interval.

Whole body malondialdehyde (MDA) concentrations, an indicator for oxidative stress, were significantly lower in the 6,600 μatm, 12,800 μatm and 24,400 μatm groups when compared to the control (Fig. 5a, Tukey HSD, p < 0.05). Additionally, MDA values of the 24,400 μatm treatment were significantly lower than values measured in 1,500 μatm and 2,900 μatm animals.

Slopes of log shell free dry mass vs. log shell width linear regressions (900–24,400 μatm) were not significantly different between treatments (F_(5,467)_ = 0.49, p = 0.7851, Fig. 5b), while y-intercepts were significantly different (F_(5,472)_ = 32.5, p < 0.0001). It appears that y-intercepts of all treatment levels (1,500–24,400 μatm) were significantly lower (no overlap in 95% confidence intervals) than that of the control group (900 μatm), indicating reduced body condition at elevated seawater *p*CO_2_ (Fig. 5c). Slopes of control (900 μatm) and field animals collected just prior to the experiment were similar (F_(1,110)_ = 0.15, p = 0.7021). Y-intercepts were significantly different between these two groups (F_(1,111)_ = 17.94, p < 0.0001), with slightly reduced condition in the control animals vs. field collected animals (Supplementary Table S5).

*M. arenaria* and *M. balthica* remained burrowed during the entire experimental duration in all treatments and no mortality was observed for these two species.

### Meiofauna community response

**Figure 6:**
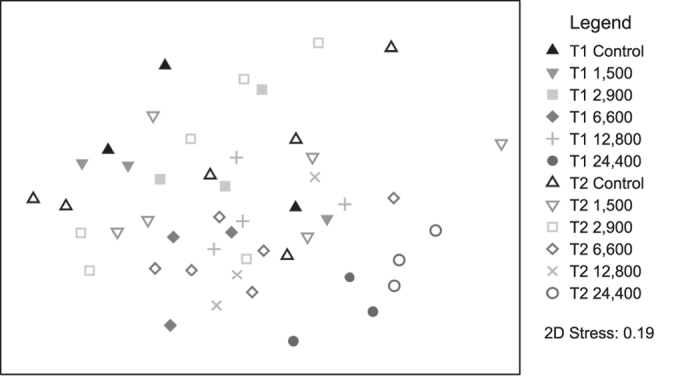
MDS plot representing the meiofauna community composition. Data based on square root transformed densities. Symbols indicate the factors “time” (T1 = after six weeks and T2 = after twelve weeks) and “treatment”. (Control, 1,500 µatm–24,400 µatm).

A total of twelve meiofauna groups were found. The four most abundant taxa were Nematoda (72.5 ± 7.5%), Gastrotricha (11.0 ± 7.1%), Copepoda (5.9 ± 4.5%), and crustacean nauplii (2.6 ± 3.2%). Total meiofauna densities ranged from 218 to 988 ind. 10 cm^−^^2^. Towards the end of the experiment, significantly higher total meiofauna densities were found in the 24,000 μatm treatment compared to all other treatments, except for the 1,500 and 2,900 μatm treatment (Pairwise tests (Treatment * Time), p(MC) ≤ 0.0495, Supplementary Table S6a). Meiofauna community composition significantly differed based on the factors treatment (Main test, p(perm) = 0.0004, PermDisp < 0.05) and time (Main test, p(perm) = 0.0022). The meiofauna community of the 24,400 μatm treatment had a significantly different composition compared to the assemblages in all other treatments (p(perm) ≤ 0.0201, Fig. 6, Supplementary Table S6a). Evenness and diversity of the total meiofauna community decreased with time (p(perm) ≤ 0.0275, PermDisp < 0.05) but did not differ between treatments.

The densities of nematodes (352.43–1733.64 ind. 10cm^−^^2^), did not consistently differ over time or between treatments. It only occasionally differed between the 6,600 μam and 1,500 μatm treatment after six weeks, and between the 24,400 μatm and 12,800 μatm treatment after twelve weeks (see Supplementary Table S6a). A total of 36 nematode genera were found. The overall most abundant genera (>5%) were *Ascolaimus* (34.7% ± 2.2%), *Metachromadora* (13.2% ± 1.0%), *Hypodontolaimus* (9.4% ± 1.0%), *Microlaimus* (5.9% ± 0.5%) and *Enoplolaimus* (5.6% ± 0.6%). Multivariate analyses of relative and total densities of nematode genera did not reveal any significant difference between samples according to the *p*CO_2_ level, time or a combination of both. Samples exhibited an overall high evenness (Pielou’s evenness index) ranging from 0.60 to 0.89 but univariate measures based on densities and relative abundances (genus richness, Shannon-Wiener index, Simpson index, Pielou’s evenness index) did not differ between samples based on the factors treatment, time or a combination of both.

**Figure 7:**
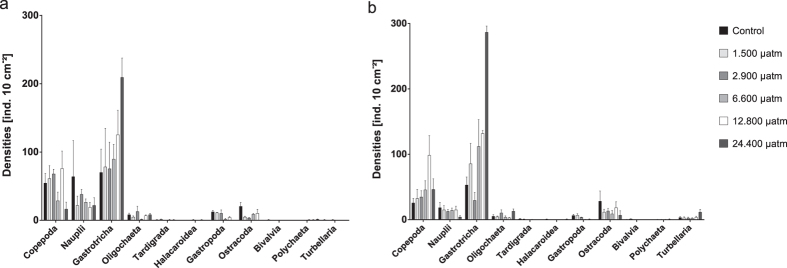
Average meiofauna densities excluding nematodes. Sampled after (**a**) six weeks and (**b**) twelve weeks plotted per treatment in number of individuals per 10 cm^2^ (mean ± standard error).

When excluding Nematoda from the community composition analysis, Permanova revealed significant differences between treatments (p(perm) = 0.0002, PermDisp < 0.05) and over time (p(perm) = 0.0023). Differences occurred between the 24,400 μatm treatment and all other treatments (p(perm) ≤ 0.0298). According to the SIMPER analysis, Gastrotricha contributed most to these differences (25.53–41.08%), followed by Copepoda (24.19–11.25%), Ostracoda (14.98–9.88%) and Gastropoda (11.73–7.40%). Gastrotricha, Copepoda, Nauplii and Ostracoda contributed most to the difference over time (22.40%, 18.70%, 15.47% and 11.77%, respectively). Univariate analyses of the densities of the taxa contributing most to the differences (Fig. 7) revealed diverse responses. Copepoda densities were not impacted by treatment and time, whereas nauplii densities decreased significantly from the first to the second sampling time point (p(perm) = 0.0246). Gastrotricha densities were increased in the 24,400 μatm treatment compared to the control, 2,900 μatm and 6,600 μatm treatment (Pairwise test, p(perm) ≤ 0.0305) and densities of the 12,800 μatm treatment were increased compared to the control and 2,900 μatm group (p(perm) = 0,0171 and p(perm) = 0,034, respectively) but did not differ from the 1,500 μatm and 6,600 μatm treatment. Ostracoda densities increased over time (Main test, p(perm) = 0.0385) but were significantly lower in the 24,400 μatm treatment compared to the control, 1,500 μatm and 6,600 μatm treatment (p(perm) ≤ 0.0163). Gastropoda densities decreased over time (p(perm) = 0.0001, permDisp < 0.05) and differed between treatments (p(perm) = 0.0097) with decreased densities in all treatments ≥6,600 μatm (p(perm) ≤ 0.0269) when compared to treatments ≤ 2,900 μatm. At six weeks, Turbellaria were absent in >80% of the samples, while after twelve weeks densities ranged between 1 and 9 individuals per sample with a presence in >50% of all samples.

### Bacterial community

Community fingerprinting of benthic bacterial communities by ARISA indicated similar average OTU numbers for all treatments and time points, i.e. for 6 and 12 weeks respectively 196 ± 9 and 179 ± 14 (900 μatm), 191 ± 13 and 196 ± 8 (1,500 μatm), 190 ± 15 and 174 ± 17 (2,900 μatm), 188 ± 12 and 179 ± 19 (6,600 μatm), 184 ± 17 and 183 ± 13 (12,800 μatm), and 184 ± 11 and 189 ± 12 (24,400 μatm). The percentage of shared OTUs among the different EUs for a given treatment and time could be as low as 53–70% (max. 60–78%), and varied between 70–83% for joint OTU profiles (one per treatment and time point). NMDS did not show a separation of bacterial communities according to CO_2_ treatment and/or time (data not shown). However, PAST analyses indicated a significant difference between bacterial communities thriving at 900 μatm (control) and 24,400 μatm (abundance data only, Bonferroni-corrected *p* < 0.05 after 6 and 12 weeks, group separation R < 0.5, Supplementary Table S7), as well as between 1,500 μatm and 24,400 μatm (abundance and presence-absence data, Bonferroni-corrected *p* < 0.05 after 6 and 12 weeks, group separation R < 0.5). Variation partitioning demonstrated that *p*CO_2_ treatment and time had both significantly influenced bacterial community composition, together explaining 11.7% of the observed variation (*p* < 0.001). Time alone explained 5% (*p* < 0.001) of the community changes and *p*CO_2_ treatment 6.9% (*p* < 0.011). The interaction between the two factors was not significant (*p* > 0.05).

## Discussion

To our knowledge, this is the first experiment to examine the impact of elevated seawater *p*CO_2_ on benthic infaunal communities in coastal sandy sediments from the Baltic Sea under near-natural conditions. Our study indicates that persistent (3 month) strong leakage from a sub-seabed CCS site and the subsequent distribution of a high *p*CO_2_ bottom water plume could lead to massive accumulation of *C. edule* on the seafloor and strong reductions in survival, with a high sensitivity especially for smaller bivalve size classes. Changes in microbial and meiofaunal community composition at the highest *p*CO_2_ level indicate a direct or cascading effect of elevated CO_2_ concentration on the entire benthic assemblage in a coastal sandy sediment ecosystem. However, we also demonstrate pronounced sub-lethal effects at lower treatment levels that warrant further research attention.

### High *p*CO_2_ changes coastal bivalve communities and bivalve behaviour

The observed behavioural changes of *C. edule* as a result of exposure to high seawater CO_2_ correspond well with responses observed for the same species during exposure to hypoxia^70^,^71^. With increasing *p*CO_2_, moribund or weakened *C. edule* accumulated on the sediment surface. The fraction of *C. edule* on the surface of the sediment increased with duration of the experiment. Behavioural responses such as gathering of bivalves on the seafloor could be used as cost-efficient tool for future monitoring of sub-seabed CCS storage sites, e.g. by towing camera systems across large sea floor areas, when appropriate software processing and detection tools are being developed. Quite similarly, Widdicombe *et al*.^23^ observed emersion of infauna echinoderms from the sediment during a high – CO_2_ mesocosm incubation when pH values dropped below 6.5.

We observed substantial mortality of smaller size classes of *C. edule* in all treatments >6,000 μatm. This corresponds with previous work on other bivalve species where smaller juveniles appeared to be more sensitive to elevated seawater *p*CO_2_^72^,^73^. This effect might be related to less favourable area to volume ratios, as smaller animals have to protect a relatively larger surface area from acid-base disturbance and relatively larger shell area from (internal) dissolution. Shell production costs and inorganic carbon demand for calcification are also much higher in smaller bivalves^31^,^74^. Size-dependent mortality implies that leakage could lead to an alteration of the demographic structure of bivalve communities.

In our study, shell corrosion was evident from 1,500 μatm to 24,400 μatm and signs of severe dissolution and presence of holes were found in most animals exposed to 12,800 μatm and 24,400 μatm. These findings indicate that even moderate degrees of acidification can already lead to non-reversible shell damage. Outer shell corrosion has been observed in a number of gastropod and bivalve molluscs^34^,^75^ and has often been linked to absence of a thick and intact periostracum^34^. *C. edule* is characterized by a very thin periostracum (ca. 2 μm)^76^ and a shell that is exclusively composed of aragonite, the polymorph of calcium carbonate that is most prone to dissolution^50^,^77^. In contrast, mytilids are protected by a periostracum of >20 μm^76^ and can live in seawater which is strongly undersaturated for calcium carbonate (Ω_arag_ < 0.2) as long as their periostracum is intact. Corrosion starts to occur when the periostracum is mechanically damaged^35^, leading to complete dissolution of the shell in extreme cases, e.g. in deep-sea hydrothermal vent mussels that live in strongly acidic waters with pH <6^33^. While bivalves are able to repair holes and fractures in their shells^78^, it is apparent from our results that *C. edule* in the highly acidified treatments were unable to allocate sufficient resources to shell repair, or that the rate of dissolution simply overwhelmed their repair capacity. Holes in their shells probably lead to massive and energy intensive stimulation of the immune system due to invasion of foreign microorganisms and loss of valuable proteins from the extrapallial fluid, the fluid which is in contact with the inner side of the shell^79^. In summary, progressive shell corrosion, even in the lower treatment levels (i.e. 1,500 μatm and higher), constitutes a large problem as shell integrity is essential for fitness of bivalve species.

One of the toxic effects of elevated oxyradical formation in cells is an increase in lipid peroxidation levels^80^. A commonly employed mode of detection is the reaction of lipid peroxidation intermediates with thiobarbityric acid in the so called TBARs assay. One of the major products of lipid peroxidation, but by far not the only one, is malondialdehyde (MDA), which serves as a marker for oxidative stress^81^. Oxidative stress is frequently also related to metabolism^82^,^83^. Previous studies on bivalves suggest a good relationship between high MDA accumulation and elevated metabolic rate^84^,^85^. MDA concentration was low in bivalves exposed to *p*CO_2_>6,600 μatmsuggesting that animals exposed to such high CO_2_ concentrations suffer from metabolic depression. While intermediate levels of acidification (i.e. <4,000 μatm) have repeatedly been shown to cause elevated metabolic rates^86^,^87^ and increased oxidative stress^32^ in a number of bivalve species, studies using treatment levels of >4,000 μatm have generally found metabolic reduction^86^,^88^.

When employed as a strategy in the long run, metabolic suppression will lead to consumption of endogenous energy stores and reduced fitness. It has been demonstrated in a range of marine invertebrate species that acidification primarily impacts energy allocation processes and that ultimately, sensitivity is defined by depletion of available energy (scope for growth) by basal metabolism or reduced energy uptake^17^,^89^. In support of this view, all high-CO_2_ exposed *C. edule* in our experiment (1,500–24,400 μatm) were characterized by a reduction in body condition, as indicated by reductions in shell-free dry mass in relation to shell width. This effect becomes more pronounced at the higher treatment levels (Fig. 5b,c) and correlates with reduced MDA accumulation. These findings indicate a negative scope for growth, which is unsustainable in the long - run. In a next step, it will be important to investigate aerobic metabolism and energy uptake in this species to better understand the underlying processes leading to energy budget disturbance.

*M. arenaria* and *M. balthica* survived the complete experimental duration. Even though abundance of these two species was low, the observations confirm our original hypothesis that *C. edule*, the species most sensitive to hypoxic stress, also is the most vulnerable to ocean acidification^90^. *M. balthica* is a species which is generally quite tolerant towards stressful conditions. It occurs deep in the sediment where oxygen availability is low^91^ and tolerates polluted sediments^54^. Resistance of *M. arenaria* could be due to a thick, protective periostracum (20 μm)^76^.Additionally, a greater burrowing depth^92^ might render this species less sensitive to acidified seawater. All bivalves used in this study have a shell consisting of aragonite, the more soluble calcium carbonate polymorph^50^,^76^. While the mineralogy is an important factor in defining susceptibility of bivalves^34^, there is a high variation in vulnerability between species with the same shell mineralogy^30^. Crystal size and the proportion of organic matrix within the shell play an additional factor in resistance against environmental stress such as acidified seawater^76^.

### Very high pCO_2_ induces shifts in meiofaunal community structure

The findings of this experiment indicate that when very high seawater carbon dioxide levels occur, meiofauna community structure at higher taxon level can change as a result of differential sensitivity. The changes in densities were most severe in some of the less abundant meiofauna taxa that are not often reported. While most acidification studies focused on the dominant taxa such as Copepoda and Nematoda^23^,^25^,^37^,^39^, only few other studies emphasized the importance of changes in taxon composition, particularly including less abundant taxa as a proxy for the status of benthic environments^93^,^94^,^95^.

In this study, densities of three meiofauna taxa were affected by changing *p*CO_2_ (i.e. Gastrotricha, Gastropoda, and Ostracoda). In accordance with the results of the macrofauna analysis, the calcifying Gastropoda and Ostracoda suffered from decreased densities in treatments with high *p*CO_2_ (at >6,600 μatm and >24,000 μatm, respectively). Densities of Gastrotricha on the other hand increased significantly at the highest treatment level. It is, however, impossible to differentiate whether this is the direct result of the seawater acidification having a positive effect on their physiology (growth and reproduction) or whether this group was favoured by indirect factors, such as changes in food availability or space occupation by the disappearance of macrofauna or other taxa. Gastrotricha, as well as many other meiofauna taxa, feed on bacteria and protozoa^96^. The divergent microbial community in the highest acidification treatment could be the driver of the change in meiofaunal community structure with a strong increase in Gastrotrich densities. Despite all uncertainties, our study shows that Gastrotricha act as a physiologically tolerant, opportunistic taxon, apparently benefitting from severe seawater acidification.

In a laboratory experiment without macrofauna, Kurihara *et al*.^39^ found no differences in the abundance and biomass of nematodes, harpacticoid copepods or harpacticoid nauplii between treatments with 380 ppm CO_2_ (control) and 2,400 ppm CO_2_ (acidified) over a time frame of 56 days. Several single-species studies reported decreased reproduction and slowed larval development of copepods when seawater *p*CO_2_ is elevated^97^,^98^,^99^. Interestingly, the decline in densities of nauplius larvae over time in our mesocosm experiment occurred both in the control and acidified treatment indicating that the reproductive success of the copepods was not influenced by increased pCO_2_.

Nematodes, the most abundant taxon in our experiment, seemed to be unaffected by the different treatments in terms of community composition and abundance with no significant changes over time. Several experimental studies on nematodes indicate that a certain threshold exists above which nematodes do not suffer from pH reduction in short- to medium-term (days to weeks) experiments. Once pH drops below this threshold (~pH 6), a decline of nematode densities has been observed that can be assigned to direct physiological responses^40^. Widdicombe *et al*.^23^ found significant changes in nematode community structure only after a 20 weeks exposure and only at pH values lower than 6.0, while macrofauna decreased in abundance at much less severe pH changes. Similarly, in a different mesocosm experiment, nematodes remained unaffected following a seven week exposure to seawater pH of 7.5^25^. In the study of Takeushi *et al*.^40^, nematode survival was monitored over a time frame of one week and only in the most severe treatments with a pH of 5.4 and 5.1 survival rates were significantly decreased with a species specific response related to the activity of the nematodes. Sub-lethal measures such as reproductive success and scope for growth might be more appropriate variables to assess sensitivity during medium-term exposure^39^. In our case nematode densities remained stable in all treatments and between time steps, suggesting that reproduction had most likely taken place. This was supported by the occurrence of juveniles and gravid nematodes.

### High *p*CO_2_ impact on bacterial communities

Significant differences between bacterial communities became evident when comparing the highest treatment (24,400 μatm, severe leakage scenario) to the control (900 μatm) and slightly increased CO_2_ level (1,500 μatm). This suggests the presence of bacteria adapted to a wide range of natural fluctuations in *p*CO_2_, but also the risk of substantially influencing ecosystem functionality at very high *p*CO_2_. Part of the potentially occurring changes in bacterial community structure might have been masked by the high spatial variability of the bacterial communities, as suggested by low R-values (PAST analyses) and a low percentage of shared OTUs among the EUs of a given treatment and time. The overall average number of OTUs in this study (186 ± 14 OTUs, 0–1 cm sediment depth, all treatments and time points) was slightly higher than the average value obtained from samples taken during a previous study on shallow subtidal sands in the North Frisian Wadden Sea^100^ (145 ± 45.7 OTUs, 0–5 cm sediment depth, different seasons). Both *p*CO_2_ level and time played a significant role in the observed community shifts, but as the two factors did not significantly interact, at this point, the prolonged *p*CO_2_ treatment can neither be confirmed nor disregarded as a causal factor. Overall, influential factors might, besides a direct CO_2_ influence, include a change in sediment nutrient composition or a change in sediment bio-irrigation rates mediated by dying cockles. While *C. edule* has previously been shown to significantly influence the microphytobenthic primary production due to release of NH_4_^+53^, bacterial abundance was not significantly influenced by bio-diffusing activities of *C. edule*. The comparatively minor effects of substantial decrease in *C. edule* density at the highest CO_2_ treatment level on the microbial community structure could be explained by a less pronounced impact of *C. edule* on sediment oxygen and nutrient fluxes compared to polychaetes (*Nereis diversicolor*) that construct elaborate burrows and strongly shape microbial communities^101^. However, the loss of an important ecosystem engineer poses unknown risks. A progressive loss of *C. edule* could affect settlement success of other macrobenthic species (e.g. polychaetes) that are otherwise competitively impacted by *C. edule*^52^. These species could more strongly impact microbial and meiofauna communities or alter ecosystem functionality during long-term CO_2_ leakage. Similar to findings in our experiment, Kerfahi *et al*.^46^ demonstrated shifts in sediment microbial communities (top 2 cm of the sediment) along CO_*2*_ clines (600–1,600 μatm) in the Mediterranean. The authors found abundances of most dominant genera to be unaffected by CO_2_, while 5% of genera differed in abundance along the CO_2_ cline. Whether these changes can alter the biogeochemical functions of marine sediments still needs to be investigated in future experiments.

We can conclude that experimental medium-term exposure to high seawater *p*CO_2,_ which mimics strong leakage from sub-seabed CCS sites, can cause high mortality of *C. edule* and shifts in the composition of meiofauna and microbiota in a shallow sandy sediment environment. The response of bivalves was strongly species-specific and size-dependent. *C. edule* was found to be sensitive to most treatment levels with signs of shell corrosion at *p*CO_2_ ≥1,500 μatm and reduced body condition at CO_2_ ≥1,500 μatm, which points at a negative, unsustainable energy budget. This is supported by reduced tissue MDA accumulation at ≥6,600 μatm. Thus, even lower levels of leakage resulting in seawater *p*CO_2_ values of >1,500 μatm could strongly impact *C. edule* dominated sediments in the long - run as they might lead to reduced growth, enhanced mortality and reduced reproductive output. Longer – term ecological studies on *C. edule* are needed to investigate these processes in more detail. The dominant meiofauna taxa tolerated the highest acidification levels. Rare taxa on the other hand were affected by very high *p*CO_2_ illustrating a decrease in calcifying organism abundance and an increase in other, more opportunistic taxa. While the calcifying organisms where likely directly affected by seawater acidification, it is unclear whether the response of other taxa is directly related to acidification or rather a result of cascading effects due to high*C. edule* mortality. Severe leakage from storage sites that result in very high seawater *p*CO_2_ (>20,000 μatm) on a longer time scale could thus lead to significant changes in the overall benthic community structure, with unforeseen consequences for ecosystem health and functions. Lower levels of leakage (<5,000 μatm) have sub – lethal impacts on the dominant macrofauna species and may impact benthic ecosystem function in the long – term. Clearly, more research effort needs to be devoted into studying longer term consequences of exposure to lower seawater and sediment *p*CO_2_ levels to better constrain the risks that are associated with sub – lethal species responses, particularly with respect to potential impacts on sediment meiofauna and bacterial communities.

## Additional Information

**How to cite this article**: Schade, H. *et al*. Simulated leakage of high *p*CO_2_ water negatively impacts bivalve dominated infaunal communities from the Western Baltic Sea. *Sci. Rep.* **6**, 31447; doi: 10.1038/srep31447 (2016).

## Supplementary Material

Supplementary Information
