# Supplementary material for: Simulated leakage of high pCO2 water negatively impacts bivalve dominated infaunal communities from the Western Baltic Sea
Source: Sci Rep. 2016 Aug 19;6:31447. doi: 10.1038/srep31447 (PMC4990903; doi:10.1038/srep31447)
Supplement: Supplementary Information [file srep31447-s1.doc]

**Simulated leakage of high *p*CO_2_ water negatively impacts bivalve dominated infaunal communities from the Western Baltic Sea**

Hanna Schade, Lisa Mevenkamp, Katja Guilini, Stefanie Meyer, Stanislav N Gorb, Doris Abele, Ann Vanreusel, Frank Melzner

Supplementary Tables

|  | measured (N=20) | Calculated | | | | | measured (N=15) | |
| --- | --- | --- | --- | --- | --- | --- | --- | --- |
| **Treatment** | C_T_   [µmol/kg] | pH  (NBS scale) | pCO_2_ (µatm) | TA in (mmol/ kgSW) | ΩCa | ΩAr | Rhodomonas header tank (cells/ml) | Rhodomonas ø per treatment (cells/ml) |
| 900 (±SD) | 2038.3 (±130) | 7.842 | 923.69 (± 56,92) | 2,032.43 | 0.86 | 0.510 | 4277.30 (± 635.4) | 1938.51 (± 946.34) |
| 1,500 (±SD) | 2100.3 (±157.7) | 7.654 | 1,461.88 (± 190,91) | 2,049.95 | 0.57 | 0.340 | 4401.17 (± 413.5) | 2111.42 (± 921) |
| 2,900 (±SD) | 2170.2 (±126.7) | 7.360 | 2,882.51 (± 352,93) | 2,028.44 | 0.30 | 0.170 | 3652.86 (± 269.4) | 1564.41 (± 713.1) |
| 6,600 (±SD) | 2377.9 (±165) | 6.999 | 6,630.13 (± 759,50) | 2,020.41 | 0.13 | 0.080 | 3504.39 (± 354.2) | 1860.39 (± 817.9) |
| 12,800 (±SD) | 2638.1 (±258.4) | 6.696 | 12,783.49 (± 1,549,93) | 1,936.86 | 0.06 | 0.036 | 3626.80 (± 526.7) | 2915.47 (± 2397.4) |
| 24,400 (±SD) | 3188.2 (±195.8) | 6.396 | 24,381.04 (± 2,060.83) | 1,848.87 | 0.03 | 0.017 | 4260.89 (± 467.9) | 3554.18 (± 2170.7) |

**Supplementary Table S1:** Environmental parameters

| **Supplementary Table S2:** Test for the behaviour of *C.edule.* | | | | |
| --- | --- | --- | --- | --- |
|  | df | SS | % explained variance | p(perm) |
| Treatment | 5 | 306749 | **62.9** | **0.0001** |
| Time | 27 | 33187 | **0.68** | **0.0001** |
| EU (Treatment) | 30 | 1852,8 | **0.04** | **0.0001** |
| Treatment*Time | 135 | 130160 | **26.7** | **0.0001** |
| Total | 1007 | 487420 |  |  |

**Supplementary Table S3:** Main test for the cumulative mortality and shell dissolution of *C.edule,* Kruskal-Wallis test

|  | Main test | | | |
| --- | --- | --- | --- | --- |
|  | Factor | df | X² | p |
| Cumulative mortality of *C. edule* | Treatment | 5 | 28,81 | **0,00** |
| % dissolved shells of *C. edule* | Treatment | 5 | 26,75 | **0,00** |

**Supplementary Table S3a:** Kruskal multiple comparison test of mortality of *C.edule* (Kruskal Wallis Analysis, p-value= 0.0000, critical difference 17.85412)

| **Treatment** | 900 µatm | 1,500 µatm | 2,900 µatm | 6,600 µatm | 12,800 µatm | 24,400 µatm |
| --- | --- | --- | --- | --- | --- | --- |
| 900 µatm |  |  |  |  |  |  |
| 1,500 µatm | 4.3333 |  |  |  |  |  |
| 2,900 µatm | 0.1667 | 4.5000 |  |  |  |  |
| 6,600 µatm | 11.5833 | 15.9167 | 11.4167 |  |  |  |
| 12,800 µatm | 14.2500 | **18.5833** | 14.0833 | 2.6667 |  |  |
| 24,400 µatm | **22.3333** | **26.6667** | **22.1667** | 10.7500 | 8.0833 |  |

**Supplementary Table S3b:** Kruskal multiple comparison test of shell dissolution of *C.edule* (Kruskal Wallis Analysis, p-value=0.0000, critical difference: 17.85412)

| **Treatment** | 900 µatm | 1,500 µatm | 2,900 µatm | 6,600 µatm | 12,800 µatm | 24,400 µatm |
| --- | --- | --- | --- | --- | --- | --- |
| 900 µatm |  |  |  |  |  |  |
| 1,500 µatm | 0.000 |  |  |  |  |  |
| 2,900 µatm | 5.8333 | 5.8333 |  |  |  |  |
| 6,600 µatm | 13.5000 | 13.5000 | 7.6667 |  |  |  |
| 12,800 µatm | **18.3333** | **18.3333** | 12.5000 | 4.8333 |  |  |
| 24,400 µatm | **22.3333** | **22.3333** | 16.5000 | 8.8333 | 4.0000 |  |

**Supplementary Table S4:** Mortality between different size classes of *C.edule* (Kruskal Wallis Analysis), 2 size classes: small (0-1 cm), large (1-2.5 cm)

| **Treatment** | df | chi-squared | p-value |
| --- | --- | --- | --- |
| 900 µatm | 1 | 0,01 | 0,93 |
| 1,500 µatm | 1 | 0,04 | 0,85 |
| 2,900 µatm | 1 | 1,13 | 0,29 |
| 6,600 µatm | 1 | 6,68 | **0,01** |
| 12,800 µatm | 1 | 8,77 | **0,00** |
| 24,400 µatm | 1 | 8,37 | **0,00** |

**Supplementary Table S5:** Regression of shell free dry weight (log data). The y-intercept of the Fjord control is slightly lower compared to the experimental control. As the experiment was carried out in winter (T=4.3-8.9°C) and it is known that the condition of *C. edule* decreases in winter (e.g. Newell, R. I. E., Bayne, B. L. Seasonal changes in the physiology, reproductive condition and carbohydrate content of the cockle *Cardium (=Cerastoderma) edule* (Bivalvia: Cardiidae). Marine Biology. 56, (1), 11-19 (1980)) the slightly different condition could be due to winter conditions during the experiment.

| Treatment | Equation | r² | N |
| --- | --- | --- | --- |
| 900 µatm | Y = 2,994*X - 2,009 | 0,973 | 94 |
| 1,500 µatm | Y = 2,891*X - 2,058 | 0,9553 | 136 |
| 2,900 µatm | Y = 2,978*X - 2,074 | 0,962 | 91 |
| 6,600 µatm | Y = 2,917*X - 2,079 | 0,9492 | 64 |
| 12,800 µatm | Y = 2,965*X - 2,174 | 0,9538 | 66 |
| 24,400 µatm | Y = 2,995*X - 2,269 | 0,9543 | 28 |
| Fjord Control | Y = 2,879*X - 1,959 | 0,981 | 37 |

**Supplementary Table S5a:** Test for Effect of Replicates (Nested ANOVA, Replicate nested in CO_2_ Treatment)

|  | Df | SumSq | MeanSq | F value | Pr(>F) |
| --- | --- | --- | --- | --- | --- |
| level:Replicate | 6 | 0.788 | 0.13138 | 0.4556 | 0.8411 |

| 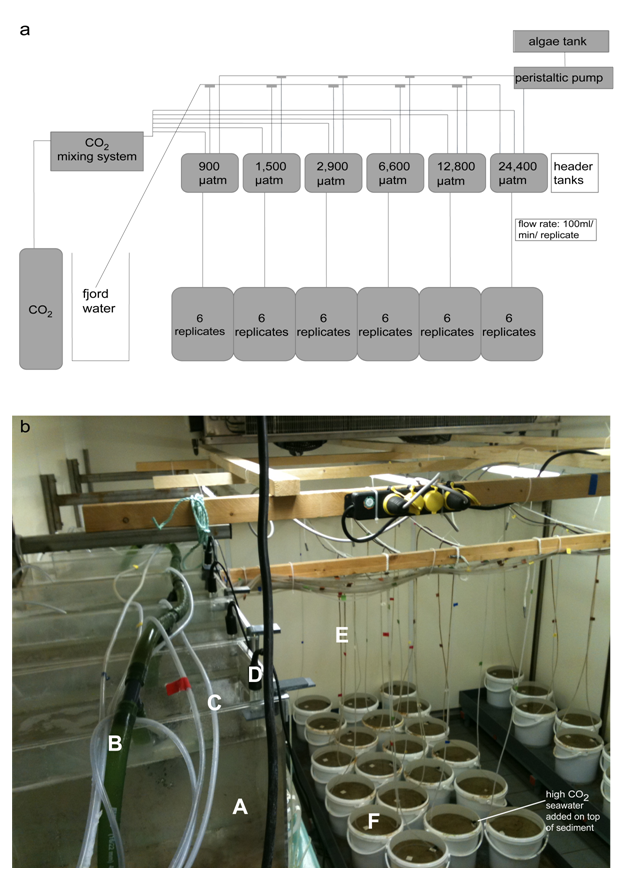 |
| --- |

**Supplementary Figure S5.** (a) Schematic illustration of the experimental setup. (b) Photographic view of the experimental setup; In a temperature controlled climate chamber at GEOMAR, fjord water (B) and algae (*Rhodomonas sp.*) (C) were pumped  into header tanks (A) supplying (E) the experimental units (F, six per CO_2_ treatment). Six different CO_2_ levels were generated through pH controlled (D = pH electrode) CO_2_ addition (IKS Aquastar).

**Supplementary Table S6:** Main test of meiofaunal densities and community composition (square root transformed) as well as nematode community composition (relative abundance and calculated densities)

|  |  | Main test | | | | |
| --- | --- | --- | --- | --- | --- | --- |
|  |  | df | SS | MS | pseudo-F | p(perm) |
| Total meiofauna | Treatment | 5 | 683.07 | 136.61 | 1.8839 | 0.1316 |
|  | Time | 1 | 54.98 | 54.98 | 3.9096 | 0.0674 |
|  | EU(Treatment | 25 | 2081.80 | 83.27 | 5.9214 | **0.0008** |
|  | Treatment*Time | 5 | 475.15 | 95.03 | 6.7575 | **0.0033** |
|  | Residuals | 12 | 168.76 | 14.06 |  |  |
|  | Total | 48 | 2870.70 |  |  |  |
| Meiofauna community composition | Treatment | 5 | 3164.10 | 632.82 | 3.0099 | **0.0004** |
|  | Time | 1 | 864.39 | 864.39 | 6.5242 | **0.0022** |
|  | EU(Treatment | 25 | 5613.90 | 224.56 | 1.6949 | **0.0254** |
|  | Treatment*Time | 5 | 1134.10 | 226.82 | 1.7119 | 0.0633 |
|  | Residuals | 12 | 1589.90 | 132.49 |  |  |
|  | Total | 48 | 11586.00 |  |  |  |
| Meiofauan excl. Nematoda | Treatment | 5 | 7577.60 | 1515.50 | 3.4291 | **0.0002** |
|  | Time | 1 | 2189.80 | 2189.80 | 6.5072 | **0.0023** |
|  | EU(Treatment | 25 | 11534.00 | 461.36 | 1.3710 | 0.0979 |
|  | Treatment*Time | 5 | 1872.70 | 374.53 | 1.1129 | 0.3744 |
|  | Residuals | 12 | 4038.30 | 336.52 |  |  |
|  | Total | 48 | 27137.00 |  |  |  |
| Nematoda | Treatment | 5 | 644.37 | 128.87 | 1.7314 | 0.1700 |
|  | Time | 1 | 125.05 | 125.05 | 6.3176 | **0.0255** |
|  | EU(Treatment | 25 | 2112.20 | 84.49 | 4.2682 | **0.0054** |
|  | Treatment*Time | 5 | 521.50 | 104.30 | 5.2692 | **0.0054** |
|  | Residuals | 12 | 237.53 | 19.79 |  |  |
|  | Total | 48 | 2882.60 |  |  |  |
| Nauplii | Treatment | 5 | 3376.20 | 675.25 | 1.1862 | 0.3039 |
|  | Time | 1 | 3485.40 | 3485.40 | 4.0269 | **0.0246** |
|  | EU(Treatment | 25 | 12868.00 | 514.73 | 0.5947 | 0.9204 |
|  | Treatment*Time | 5 | 2662.20 | 532.44 | 0.6152 | 0.8307 |
|  | Residuals | 12 | 10386.00 | 865.54 |  |  |
|  | Total | 48 | 30551.00 |  |  |  |
| Gastrotricha | Treatment | 5 | 7785.80 | 1557.20 | 4.0947 | **0.0054** |
|  | Time | 1 | 46.18 | 46.18 | 0.2334 | 0.7109 |
|  | EU(Treatment | 25 | 10346.00 | 413.85 | 2.0914 | 0.0664 |
|  | Treatment*Time | 5 | 1414.90 | 282.99 | 1.4301 | 0.2666 |
|  | Residuals | 12 | 2374.60 | 197.89 |  |  |
|  | Total | 48 | 23926.00 |  |  |  |
| Ostracoda | Treatment | 5 | 9296.40 | 1859.30 | 3.5072 | **0.0098** |
|  | Time | 1 | 1884.40 | 1884.40 | 4.4909 | **0.0385** |
|  | EU(Treatment | 25 | 13762.00 | 550.47 | 1.3119 | 0.2732 |
|  | Treatment*Time | 5 | 1292.60 | 258.52 | 0.6161 | 0.7429 |
|  | Residuals | 12 | 5035.20 | 419.60 |  |  |
|  | Total | 48 | 32944.00 |  |  |  |
| Gastropoda | Treatment | 5 | 17720.00 | 3544.00 | 14.0640 | **0.0001** |
|  | Time | 1 | 2388.10 | 2388.10 | 8.7587 | **0.0097** |
|  | EU(Treatment | 25 | 6204.90 | 248.20 | 0.9103 | 0.6059 |
|  | Treatment*Time | 5 | 1489.20 | 297.83 | 1.0923 | 0.4049 |
|  | Residuals | 12 | 3271.80 | 272.65 |  |  |
|  | Total | 48 | 34616.00 |  |  |  |
| Nematode composition (rel.abu.) | Treatment | 1 | 679.42 | 679.42 | 1.4587 | 0.1859 |
|  | Time | 5 | 2327.20 | 465.44 | 0.6278 | 0.9112 |
|  | EU(Treatment | 12 | 8896.50 | 741.37 | 1.5917 | **0.0309** |
|  | Treatment*Time | 5 | 2889.90 | 577.97 | 1.2409 | 0.2076 |
|  | Residuals | 12 | 5589.10 | 465.76 |  |  |
|  | Total | 35 | 20382.00 |  |  |  |
| Nematode composition (densities) | Treatment | 1 | 601.05 | 601.05 | 1.3790 | 0.2096 |
|  | Time | 5 | 2619.40 | 523.88 | 0.9790 | 0.5395 |
|  | EU(Treatment | 12 | 6421.40 | 535.12 | 1.2278 | 0.1003 |
|  | Treatment*Time | 5 | 2503.30 | 500.67 | 1.1487 | 0.2652 |
|  | Residuals | 12 | 5230.20 | 435.85 |  |  |
|  | Total | 35 | 17375.00 |  |  |  |

**Supplementary Table S6a:** Pairwise tests of the factor treatment of selected meiofauna groups. P(perm)- or, if indicated, p(MC) values are given.

| **Total meiofauna densities** | | | | | | |
| --- | --- | --- | --- | --- | --- | --- |
| **Treatment*Time after 12 weeks p(MC)** | 900 µatm | 1 500 µatm | 2 900 µatm | 6 600 µatm | 12 800 µatm | 24 400 µatm |
| 900 µatm |  |  |  |  |  |  |
| 1 500 µatm | 0.4005 |  |  |  |  |  |
| 2 900 µatm | 0.8379 | 0.5111 |  |  |  |  |
| 6 600 µatm | 0.4085 | 0.7809 | 0.5631 |  |  |  |
| 12 800 µatm | 0.5552 | 0.817 | 0.6937 | 0.9653 |  |  |
| 24 400 µatm | **0.0466** | 0.2711 | **0.0495** | **0.0442** | **0.0492** |  |
| **Nematode densities** | | | | | | |
| **Treatment*Time after 6 weeks p(MC)** | 900 µatm | 1 500 µatm | 2 900 µatm | 6 600 µatm | 12 800 µatm | 24 400 µatm |
| 900 µatm |  |  |  |  |  |  |
| 1 500 µatm | 0.7595 |  |  |  |  |  |
| 2 900 µatm | 0.361 | 0.6511 |  |  |  |  |
| 6 600 µatm | 0.9253 | 0.6896 | 0.2893 |  |  |  |
| 12 800 µatm | 0.1116 | 0.4528 | 0.9128 | **0.0396** |  |  |
| 24 400 µatm | 0.148 | 0.4959 | 0.8873 | 0.0501 | 0.8961 |  |
| **Nematode densities** | | | | | | |
| **Treatment*Time after 12 weeks p(MC)** | 900 µatm | 1 500 µatm | 2 900 µatm | 6 600 µatm | 12 800 µatm | 24 400 µatm |
| 900 µatm |  |  |  |  |  |  |
| 1 500 µatm | 0.3796 |  |  |  |  |  |
| 2 900 µatm | 0.6314 | 0.6486 |  |  |  |  |
| 6 600 µatm | 0.4157 | 0.6337 | 0.8101 |  |  |  |
| 12 800 µatm | 0.8957 | 0.4267 | 0.5744 | 0.2572 |  |  |
| 24 400 µatm | 0.1219 | 0.5905 | 0.2385 | 0.053 | **0.0369** |  |
| **Meiofauna Composition** | | | | | | |
| **Treatment** | 900 µatm | 1 500 µatm | 2 900 µatm | 6 600 µatm | 12 800 µatm | 24 400 µatm |
| 900 µatm |  |  |  |  |  |  |
| 1 500 µatm | 0.7912 |  |  |  |  |  |
| 2 900 µatm | 0.2463 | 0.3550 |  |  |  |  |
| 6 600 µatm | 0.1545 | 0.2540 | 0.0610 |  |  |  |
| 12 800 µatm | 0.1344 | 0.1921 | 0.0759 | 0.2253 |  |  |
| 24 400 µatm | **0.0038** | **0.0201** | **0.0012** | **0.0006** | **0.00265** |  |
| **Gastritricha p(MC)** | | | | | | |
| **Treatment** | 900 µatm | 1 500 µatm | 2 900 µatm | 6 600 µatm | 12 800 µatm | 24 400 µatm |
| 900 µatm |  |  |  |  |  |  |
| 1 500 µatm | 0.7626 |  |  |  |  |  |
| 2 900 µatm | 0.9857 | 0.8066 |  |  |  |  |
| 6 600 µatm | 0.2323 | 0.5180 | 0.2244 |  |  |  |
| 12 800 µatm | **0.0171** | 0.1363 | **0.0340** | 0.2933 |  |  |
| 24 400 µatm | **0.0055** | 0.055 | **0.0102** | **0.0305** | 0.0568 |  |
| **Ostracoda** | | | | | | |
| **Treatment** | 900 µatm | 1 500 µatm | 2 900 µatm | 6 600 µatm | 12 800 µatm | 24 400 µatm |
| 900 µatm |  |  |  |  |  |  |
| 1 500 µatm | 0.1943 |  |  |  |  |  |
| 2 900 µatm | 0.3824 | 0.6143 |  |  |  |  |
| 6 600 µatm | 0.3386 | 0.6997 | 0.7181 |  |  |  |
| 12 800 µatm | 0.8770 | 0.3456 | 0.7747 | 0.4917 |  |  |
| 24 400 µatm | **0.0123** | **0.0163** | 0.0615 | **0.0041** | 0.0644 |  |
| **Gastropoda** | | | | | | |
| **Treatment** | 900 µatm | 1 500 µatm | 2 900 µatm | 6 600 µatm | 12 800 µatm | 24 400 µatm |
| 900 µatm |  |  |  |  |  |  |
| 1 500 µatm | 0.8381 |  |  |  |  |  |
| 2 900 µatm | 0.5286 | 0.6678 |  |  |  |  |
| 6 600 µatm | **0.0116** | **0.0067** | **0.0079** |  |  |  |
| 12 800 µatm | **0.0269** | **0.0066** | **0.0049** | 0.1533 |  |  |
| 24 400 µatm | **0.0108** | **0.0001** | **0.0081** | 0.1837 | **0.0001** |  |

**Supplementary Table S7:** Main Test of bacterial community composition

|  | Factor | df | %varianceexplained | F | p |  |
| --- | --- | --- | --- | --- | --- | --- |
| response: Hellinger-transformed ARISA data. | Time + Treatment | 2 | 11.7 | 5,5858 | **0.001** |  |
| (R analyses) | Time | 1 | 5.0 | 4,8737 | **0.001** |  |
|  | Treatment | 1 | 6.9 | 6,3053 | 0,011 |  |
|  | Time x Treatment | 1 | 1.3 | 1,0342 | 0.38 |  |
| Bacterial diversity after 6 weeks (PAST analyses); Bray-Curtis/ abundance ; R=0.3133 (low group separation) | | | | | | |
|  | 900 | 1,500 | 2,900 | 6,600 | 12,800 | 24,400 |
| 900 µatm |  |  |  |  |  |  |
| 1,500 µatm | 0.264 |  |  |  |  |  |
| 2,900 µatm | 1 | 0.177 |  |  |  |  |
| 6,600 µatm | 0.9345 | 0.0645 | 0.675 |  |  |  |
| 12,800 µatm | 0.249 | **0.033** | 0.447 | 1 |  |  |
| 24,400 µatm | **0.03** | **0.0285** | 0.057 | 0.822 | 0.0645 |  |
| Bacterial diversity after 12 weeks (PAST analyses); Bray-Curtis/ abundance; R=0.2486 (low group separation) | | | | | | |
|  | 900 | 1,500 | 2,900 | 6,600 | 12,800 | 24,400 |
| 900 µatm |  |  |  |  |  |  |
| 1,500 µatm | 1 |  |  |  |  |  |
| 2,900 µatm | 1 | 1 |  |  |  |  |
| 6,600 µatm | 0.093 | 1 | 1 |  |  |  |
| 12,800 µatm | 0.0615 | 0.1425 | 1 | 1 |  |  |
| 24,400 µatm | **0.033** | **0.018** | 0.2055 | 0.1845 | 0.681 |  |
| Bacterial diversity after 6 weeks (PAST analyses); Jaccard/presence-absence ; R=0.2403 (low group separation) | | | | | | |
|  | 900 | 1,500 | 2,900 | 6,600 | 12,800 | 24,400 |
| 900 µatm |  |  |  |  |  |  |
| 1,500 µatm | 0,414 |  |  |  |  |  |
| 2,900 µatm | 1 | 1 |  |  |  |  |
| 6,600 µatm | 1 | 0,372 | 1 |  |  |  |
| 12,800 µatm | 0,7125 | 0,186 | 1 | 1 |  |  |
| 24,400 µatm | 0,1425 | **0,03** | **0,036** | 0,8865 | 0,108 |  |
| Bacterial diversity after 12 weeks (PAST analyses); Jaccard/presence-absence; R=0.2893 (low group separation) | | | | | | |
|  | 900 | 1,500 | 2,900 | 6,600 | 12,800 | 24,400 |
| 900 µatm |  |  |  |  |  |  |
| 1,500 µatm | 1 |  |  |  |  |  |
| 2,900 µatm | 0,8955 | 1 |  |  |  |  |
| 6,600 µatm | 0,234 | 1 | 1 |  |  |  |
| 12,800 µatm | 0,0975 | 0,5475 | 0,15 | 1 |  |  |
| 24,400 µatm | 0,0765 | **0,039** | 0,066 | 0,3075 | 0,0705 |  |
